# Serological and parasitological response in chronic Chagas patients 3 years after nifurtimox treatment

**DOI:** 10.1186/1471-2334-13-85

**Published:** 2013-02-13

**Authors:** Yves Jackson, Eric Chatelain, Anne Mauris, Marylise Holst, Qianqian Miao, Francois Chappuis, Momar Ndao

**Affiliations:** 1 Division of primary care medicine, Department of community medicine, primary care and emergency medicine, Geneva University Hospitals and University of Geneva, Geneva, Switzerland; 2 Drug for Neglected Diseases Initiative, Geneva, Switzerland; 3 Department of genetics and laboratory analysis, Geneva University Hospitals and University of Geneva, Geneva, Switzerland; 4 National Reference Center for Parasitology, Department of Medicine, Division of Infectious Diseases, Research Institute of the McGill University Health Centre, Montreal General Hospital, Montreal, Canada; 5 Division of international and humanitarian medicine, Department of community medicine, primary care and emergency medicine, Geneva University Hospitals and University of Geneva, Geneva, Switzerland

**Keywords:** Chagas disease, Trypanosoma cruzi, Diagnostic tests, Follow-up, Serology, PCR, Rapid diagnostic tests

## Abstract

**Background:**

With declining vectorial transmission, Chagas disease predominantly affects adults nowadays. The efficacy of nifurtimox in the chronic phase in adult patients is poorly known, particularly in regions where there is no risk of reinfection. Recommendations for treatment outcome assessment rely on serological follow-up. We evaluated the serological and parasitological response to nifurtimox in a cohort of adult patients three years post-treatment in Switzerland.

**Methods:**

Patients treated with nifurtimox in 2008 during a cross-sectional study in Geneva, Switzerland, were contacted for follow-up in 2011. Two ELISAs and a rapid immunochromatographic test were used to test 2008 and 2011 serum samples simultaneously. In addition, conventional and real-time PCR were performed on 2011 samples.

**Results:**

Thirty-seven (84.1%) of 44 eligible patients, predominantly female, middle-aged, Bolivians at the indeterminate stage, were enrolled. All 2011 ELISA and immunochromatographic tests were positive. Twenty-eight (75.7%) patients presented a lower optical density (OD) in 2011 compared to 2008. This OD difference was significant in both commercial (P < 0.001) and in-house (P = 0.002) ELISAs. Agreement between the two ELISAs was low (Kappa = 0.469). All patients had negative conventional PCR results but one (2.7%) was positive with real-time PCR.

**Conclusion:**

Our results highlight the inadequacy of serology for assessing response in adults, three years after treatment. In our cohort, 97.3% had results that could either indicate treatment failure or persistant humoral response despite treatment. The lack of accurate early post-treatment tests of cure prevents appropriate patients information and councelling. New follow-up tests are needed to assess treatments efficacy given the large adult population in need of antiparasitic therapy.

## Background

Chagas disease affects 8–10 million people in Latin America and up to 80,000 and 300,000 in Europe and in the United States respectively, two areas free from vectorial transmission [1,2]. With declining rates of vectorial infection, the burden of this chronic disease is now predominantly carried by adults [3]. Recent guidelines and practices in non-endemic countries extended treatment indications to adults at the indeterminate phase or with mild cardiopathy, to prevent or mitigate potentially lethal cardiac damage [4,5]. Benznidazole and nifurtimox are the only antiparasitic drugs registered for this indication. The significant side-effects and drop-out rates in adults raises safety and efficiency concerns about the large scale implementation of adult treatment programmes [6,7].

The main criterion for treatment success is return to sereonegativity against *T.cruzi*[8]. To date, most post treatment serological studies in adults have been conducted in patients treated with benznidazole [9]. This drug is frequently recommended by experts as the first line treatment due to its greater tolerance, even though no randomized trial has ever evaluated the comparative safety and efficacy of nifurtimox and benznidazole in adults [7,10]. There has been no follow-up study of nifurtimox treated patients in non endemic regions, where the persistance of a seropositive result cannot be attributed to vectorial reinfection.

Recently, rapid diagnostic tests have proved both sensitive and highly specific in screening adults for Chagas disease [11,12]. They facilitate the screening of hard-to-reach populations such as rural communities or migrants in conditions where access to laboratory is difficult. Conventional serologies should be used to confirm the diagnosis. No study has evaluated their use as a follow-up tool post-treatment. Conventional polymerase chain reaction (PCR) has been found to lack sensitivity in adults at the chronic phase of the disease when used as a first-line diagnostic test [13]. This results from the low and intermittent circulating parasitic load in adults during the chronic phase. No information is available about PCR in adults following nifurtimox treatment.

This study aimed to observe the changes in conventional and rapid serological diagnostic tests in a cohort of adults living in a non-endemic country after nifurtimox treatment. The presence of circulating *T. cruzi* detected by conventional and real-time PCR in this cohort was also examined. We found that all follow-up serological tests three years after treatment remained positive with one patient having a positive real-time PCR. It means that 97.3% of patients could not be classified as either suffering treatment failure or having persistant humoral response despite treatment.

## Methods

### Ethical statement

The protocol of this study was approved by the Ethical Board for Medical Research of the Geneva University Hospitals (protocol 11–162). Written informed consent was obtained from all participants.

### Study design and setting

This cohort study took place at the Geneva University Hospitals, which are the reference center for the screening and clinical management of Chagas disease in Geneva, Switzerland. Geneva hosts a substantial population of Latin American migrants originating from endemic areas for Chagas disease. Migrants’ communities and support groups were informed about the study.

### Study population

In 2008, we diagnosed *Trypanosoma cruzi* infection in 130 of 1012 (12.8%) adult Latin American migrants participating in a community-based cross-sectional study in Geneva, Switzerland [14]. The diagnosis was based on positive results in two different serological tests (BioMerieux Elisa cruzi® and the Biokit Bioelisa Chagas®). All participants with positive results were also tested with a rapid diagnostic test (Stat-Pak®). At that time, no PCR was performed to search for circulating *T. cruzi*. The 130 cases were at the chronic phase of the infection with 14 (11.3%) and 1 (0.8%) showing evidence of *T. cruzi* cardiac and digestive damage, respectively. Eighty-one participants started treatment with nifurtimox (10 mg/kg/day), however only 41 of these (56.2%) completed the 60-day treatment. Participants provided informed consent to have serum samples stored for future Chagas disease diagnostic studies. Sera were frozen (−20°C) upon collection and stored in a bio-bank at the Geneva University Hospitals.

### Study participants

From October 3^rd^ to November 30^th^ 2011, patients diagnosed with Chagas disease and who started nifurtimox treatment during the 2008 study and still living in Geneva (n = 44) were invited to participate. Patients were contacted by phone by the study nurse. In the event of five unanswered phone calls, an invitation letter was sent to the last recorded address. Study information, consent form and convocation letters were written in Spanish to enhance participation.

### Samples collection

A qualified nurse collected two blood samples from each participant’s cubital vein: one tube containing EDTA for PCR and one tube without anticoagulant for serological tests, the latter being centrifuged and aliquoted in three subsamples. All samples were stored at −20°C within 1 hour upon collection. One frozen serum aliquot per participant was sent to the National Reference Center for Parasitology, Research Institute of the McGill University Health Centre, Montreal General Hospital, Canada for in house ELISA and PCR testing.

### Patient characteristics

Sociodemographic characteristics were collected by the study nurse at the time of the blood sample collection. Information was cross-checked with those from the 2008 study. Clinical information on the stage of the disease, 2008 serum rapid immunochromatographic assay results and details about anti-trypanosomal therapy was extracted from the 2008 study files.

### Serological follow-up

In Geneva, the Stat-Pak® was performed on one of the serum aliquots according to the manufacturer’s recommendations. Results were recorded as positive or negative. After retrieving one 2008 serum aliquot for each participant, the 2008 and 2011 serum samples were tested with the Biokit Bioelisa Chagas® ELISA. All tests were done simultaneously using the same batch of tests and on the same machine to reduce random variability in measurements. In Montreal, all 2008 and 2011 samples were simultaneously tested with an in-house ELISA using fixed forms of *Trypanosoma cruzi* (epimastigotes, amastigotes, and trypomastigotes) as described elsewhere [15]. The cut-off value of the optical density (OD) for negativity of the Biokit Bioelisa Chagas® was 0.320, as instructed by the manufacturer and 0.4 for the in-house ELISA. Results were reported as a continuous variable for the measurement of OD values and as a categorical variable for the final result (positive versus negative). No cut-off for clinical significance was set for the interpretation of the quantitative OD value difference in absence of valid evidences and following the manufacturer’s recommendation.

### PCR assays

DNA extraction was performed from whole blood samples using QIAamp DNA Mini Kit (Qiagen, San Diego, CA) following the manufacturer’s instructions. Conventional PCR was performed as described by Ndao et al. [16]. LightCycler PCR detection and analysis systems (LightCycler 1.5, Roche Applied Science, Manheim, Germany) were used for amplification and quantification. The reaction was performed in glass capillaries. *T. cruzi* specific primers *TCZ-3 5*^′-^*TGC ACT CGG CTG ATC GTT T-3*^′^- and *TCZ-45*^′-^*ATT CCT CCA AGC AGC GGA TA-3*^′^- (Ndao et al.) [16] and probe *Tcruzi*TM*5*^′^*6FAM-TGC TGC ATC ACA CGT TGT GGT CCA—BBQ-3*^′^ (TIB MOLBIOL, Adelphia, NJ), were designed to amplify the parasitic genomic DNA. For amplification detection, the LightCycler™ Fastart TaqMan® Master Kit (Roche Applied Science) was used as recommended by the manufacturer. Briefly, a total of 20 μl of reaction consists of 2 μl each of primers TCZ-3 and TCZ-4 (1 μM final concentration), 2 μl (0.4 μM) of *Tcruzi* TM, 4 μl of 5x reaction buffer mix and 5 μl distilled H_2_O. Each sample was analysed in triplicate, and 5 μl of DNA sample was added in each capillary. The qPCR reaction was performed with the cycle condition of 1 cycle at 95°C for 10 min, 45 cycles of 95°C for 10 s, 61°C for 8 s, and 72°C for 20s and 1 final cycle of 40°C for 30 s. The cycle number, determined by the melting curve, was presented as the number of PCR cycles needed for the fluoresce signal of the amplified products to exceed the detected threshold value.

### Statistical analysis

Continuous non-normally distributed variables are presented with median and interquartile range (IQR). We used the parametric paired *t*-test and the non-parametric Wilcoxon rank sum test to compare 2008 and 2011 OD values. Fischer’s exact and McNemar’s tests were used for comparison of independent and paired proportions. The significance level was set at 0.05%. Analyses were conducted using SPSS software, version 20.

## Results

### Patients

Of the 44 eligible patients, 37 (84.1%) were enrolled in the study, while 3 (6.8%) refused to participate and 4 (9.1%) could not be reached. Table 1 shows the sociodemographic and clinical characteristics of the 37 participants. The 7 non participants had similar characteristics (data not shown).

**Table 1:** Sociodemographic and clinical characteristics of the 37 participants

		**N (%) or median (range)**
**Gender**	Male	7 (18.9)
	Female	30 (81.1)
**Age (years)**		44 (25–59)
**Origin**	Bolivia	36 (97.3)
	Argentina	1 (2.7)
**Staging**	Indeterminate	31 (83.8)
	Cardiopathy	6 (16.2)
**Duration of treatment (days)**	60	25 (67.6)
	30–59	4 (10.8)
	<30	8 (21.6)

### ELISA and rapid immunochromatographic tests

All 37 post-treatment results were positive with the two ELISAs and the rapid immunochromatographic test (Table 2). Twenty-eight (75.7%) participants had decreased ELISA OD values in 2011 compared to 2008 with significantly lower OD values in both the Biokit Bioelisa Chagas (P < 0.001) and the in-house ELISA (P = 0.002). Agreement between the two tests was low (Kappa = 0.469). There was no significant association between the proportions of patients with reduced OD value in 2011 and gender (P = 0.66), age (P = 0.45), stage of disease (P = 1) and treatment duration (P = 0.69).

**Table 2:** Results of ELISA, rapid immunochromatographic and PCR (conventional and real-time) tests in 37 patients with Chagas disease, three years after treatment with nifurtimox

	***Biokit Bioelisa Chagas***^***®***^	***In-house ELISA***	***Stat-Pak***^***®***^	***Conventional PCR***	***Real time PCR***
	***n (%)***	***n (%)***	***n (%)***	***n (%)***	***n (%)***
**Positive**	37 (100)	37 (100)	37 (100)	0 (0)	1 (2.7)
**Negative**	0 (0)	0 (0)	0 (0)	37 (100)	36 (97.3)
**Inconclusive**	0 (0)	0 (0)	0 (0)	0 (0)	0 (0)

### PCR testing

Conventional PCR testing was negative in all 37 patients, whereas real-time PCR was positive in 1 (2.7%) patient.

## Discussion

This study shows that none of two ELISAs and a rapid immunochromatographic test (Stat-Pak®) reverted to negativity three years after nifurtimox treatment in a cohort of adults at the chronic stage of Chagas disease living in an area without vectorial transmission. One patient was found positive by Real-Time PCR. Therefore, 97.3% participants could not be adequately informed about their current clinical status, with a single patient conclusively found to be still infected. The positive serological test results could either indicate treatment failure or simply reflect the window period of persistent humoral response despite potential treatment efficacy. Moreover, we found low agreement between the two ELISAs in regards to decreasing OD values post treatment. Whereas the main limitation of the study is the sample size of 37 participants, it is the largest serological follow-up study of adults treated with nifurtimox published to date and the first study carried out in a non-endemic country.

To date, there is no early marker of treatment efficacy in adults. Currently, the criterion of treatment success is the return to negativity of a previously positive serological test and it is recommended to undergo a 10- to 20-year long serological follow-up to assess treatment efficacy in adults [8]. These recommendations are more appropriate for the management of children, whose serology usually return to negativity within months. Our findings highlight the lack of utility of these recommendations for adults in clinical settings as all qualitative serological results remained unchanged 3 years after treatment with nifurtimox.

These results, in patients with no risk of vectorial reinfection following treatment, confirm the long lasting anti-*T. cruzi* humoral response after treatment, which can persist for more than 20 years [17-20]. In addition to showing this long window period, these studies in adults treated with benznidazole and nifurtimox found overall low (<50%) seronegativation rates. There is evidence that a seronegative response following benznidazole treatment is correlated with a reduced risk of cardiac damage and possibly of mortality [17,20,21]. Few studies have specifically evaluated serological response following nifurtimox treatment in adults. Fabbro and colleagues followed a cohort of 27 adults over 21 years in Argentina. They found a trend towards a higher rate of seronegativation in those treated with nifurtimox (40.5%) than with benznidazole (33.3%) [17]. Coura et al., found no serological change in 19 Brazilian patients, one year after nifurtimox treatment [18]. In our cohort, there was no way to reliably discriminate between treatment failure and latency before seronegativation, except for the single patient with a positive PCR indicating treatment failure. Moreover, the low agreement between tests regarding OD kinetic tends to demonstrate the lack of utility of this potential criterion of cure. We observed a significant decrease in optical density levels in both ELISAs tests in 75.7% patients. The exact significance of this finding is difficult to assess, but a non-randomized study performed following treatment with benznidazole showed a positive correlation between declining antibody titers and a reduction in the risk of cardiac damage [20]. In Argentina, Fabbro et al. showed that changes in antibody titers occurred 7 years or later after nifurtimox treatment [17]. The ongoing BENEFIT cohort study will provide additional information on the serological kinetic and the protective effect of benznidazole - but not nifurtimox - in adults [22].

No follow-up study has addressed the evolution of rapid diagnostic test results after treatment. In our study population, the Stat-Pak® rapid immunochromatogaphic test has proved reliable at the diagnostic stage [11]. However, after three years, all rapid tests remained positive, which is consistent with ELISA results. Therefore, the Stat-Pak® cannot be recommended as a test of cure, at least in the three year post-treatment period.

Different PCR techniques and primers are used to detect circulating *T.cruzi*. Overall, they are not appropriate to diagnose the chronic stage of infection due to limited (60-90%) sensitivity, technical challenges and lack of standardization [10,13,23]. However, PCR is useful in verifying infection where there are contradictory serological results, and to confirm treatment failure where there are persistent seropositive results following treatment. Real-time PCR is considered more sensitive for parasite detection than conventional methods and therefore may be a better tool to assess treatment failure [24]. In this study, we used two PCR techniques and found a discordant result in one (2.7%) patient and concordant negative results in all others. In the absence of pre-treatment PCR measurements, we were not able to identify changes. PCR is constantly evolving and efforts are underway to standardize methods [13]. Further studies are needed to assess their exact role as a test of cure.

The limited efficacy of currently available treatments and the lack of rapidly responsive tests of cure have a significant impact on the clinical management of adults and on public health. In our experience, the inability of clinicians to propose rapid and appropriate information on the treatment outcome soon after its completion may create some resistance in patients during the pre-treatment information session. Some of our patients have been reluctant to start a long and potentially poorly tolerated treatment in absence of a rapidly measurable outcome. Moreover, clinicians are unable to adequately inform treated patients on their subsequent risk of cardiopathy as long as the tests remain positive. This hampers personalized counseling on follow-up and forces patients to undergo serial serological tests over years. Today’s patients are highly mobile and Chagas disease has become a global health problem [25]. International migrants with Chagas disease, amounting to half a million at least, may receive treatment in one country and subsequently move to another, making the currently recommended long-term serological follow-up highly impracticable [25]. Moreover, given the frequent presence of other modifiable cardiac risk factors in adults with Chagas disease, the lack of accurate post-treatment evaluation strategies hamper clinicians in differentiating between possible causes of subsequent cardiac damage [26]. The reliance on the return to negativity of serologies as a proof of treatment success means that treated patients are denied the opportunity to donate blood and organs for many years until tests may eventually become negative. This is unfortunate as Latino American migrants are more willing to donate blood than local residents in Switzerland [14]. Finally, new treatment evaluation is made difficult in absence of early marker of cure.

## Conclusions

In conclusion, currently recommended strategies to assess treatment outcome in adults, at least in those receiving nifurtimox, are useless in clinical setting given the extremely long time-frame necessary to identify change, and rapid diagnostic tests seem not to offer a valid alternative. In addition to searching for reactive antigens, new diagnostic techniques such as proteomics, flow cytometry and the monitoring of *T. cruzi* specific T cells response are currently under investigation and will hopefully improve the early assessment of treatment outcome [27-30]. The management of adult Chagas disease needs rapid progress. More efficient and safer treatments, together with accurate and early post-treatment tests of cure, will bring about a giant leap forward in the clinical management of millions of adult patients.

## Abbreviations

PCR: Polymerase chain-reaction; ELISA: Enzyme linked immunosorbent assay; EDTA: Ethylene Diamine Triacetic Acid; OD: Optical density.

## Competing interests

The authors declare that they have no competing interests.

## Authors’ contribution

YJ conceived the study, performed the statistical analysis and wrote the manuscript. EC conceived the study, wrote the protocol, reviewed the analysis and proofread the manuscript. AM performed the serological analysis and proofread the manuscript. MH gathered the samples and proofread the manuscript. MQ performed the serologies and the PCR and proofread the manuscript. FC conceived the study and proofread the manuscript. MN performed the serologies and the PCR and proofread the manuscript. All authors read and approved the final manuscript.

## Pre-publication history

The pre-publication history for this paper can be accessed here:

http://www.biomedcentral.com/1471-2334/13/85/prepub
